# The troubling liaison between cancer and metabolic syndrome in chronic inflammatory rheumatic diseases

**DOI:** 10.1186/s13075-021-02465-3

**Published:** 2021-03-19

**Authors:** Giovanni Cioffi, Ombretta Viapiana, Luigi Tarantini, Giovanni Orsolini, Luca Idolazzi, Federica Ognibeni, Andrea Dalbeni, Davide Gatti, Angelo Fassio, Giovanni Adami, Maurizio Rossini, Alessandro Giollo

**Affiliations:** 1 Rheumatology Section, Department of Medicine, University of Verona, Verona, Italy; 2 Division of cardiac rehabilitation, San Pancrazio Hospital, Arco di Trento, Trento, Italy; 3 Rheumatology Unit, Policlinico Borgo Roma, Piazzale Scuro 10, 37134 Verona, Italy; 4 Department of cardiology, Ospedale civile S. Martino, Belluno, Italy; 5 Department of Medicine, General Medicine and Hypertension Unit, University of Verona & Azienda Ospedaliera Universitaria Integrata of Verona, Verona, Italy

**Keywords:** Cancer, Metabolic syndrome, Inflammatory autoimmune diseases, Rheumatoid arthritis, Psoriatic arthritis, Ankylosing spondylitis, Disease activity

## Abstract

**Background:**

Several studies on community populations found that metabolic syndrome (MetS) is associated with higher risk for total incident cancer with a predisposition for specific types of cancer. These findings have never been analyzed in patients with chronic inflammatory rheumatic and musculoskeletal diseases (RMD). We assessed prevalence/incidence and factors related to the development of cancer in a large cohort of these patients and evaluate whether MetS and its components were associated with cancer independent of traditional markers of inflammation.

**Methods:**

Between March 2014 and April 2016, 474 patients with RMD involved in a cardiovascular primary prevention program were consecutively recruited into this ambispective (combination of retrospective/prospective) study. They underwent clinical, laboratory, and echocardiographic evaluations. MetS was diagnosed according to the ATPIII criteria.

**Results:**

Duration of follow-up was 42 [18–60] months. Patients with a diagnosis of cancer (made before recruitment or during follow-up) were 46 (9.7%). Cancer was diagnosed in 22/76 patients (29%) with MetS and in 24/398 patients (6%, *p* < 0.001) without MetS; nearly two thirds of malignancies belonged to those traditionally related to MetS. MetS was the strongest cancer risk factor. Cancer was positively associated with the number of MetS components identified in each patient. Beyond MetS, cancer was associated to older age and increased inflammatory disease activity; this information allowed to build a simple performance indicator highly sensitive for cancer development.

**Conclusion:**

In light of our results, an increasingly accurate assessment of MetS would be required in patients with RMD as potential measure of clinical outcomes including the risk of cancer.

## Keypoints

- In subjects with chronic inflammatory RMD, the development of cancer is not uncommon and nearly two thirds of malignancies belong to those traditionally related to metabolic syndrome (MetS).
- More than a quarter of the patients in this study with MetS developed cancer overtime, so MetS represents the strongest risk factor independently of the various components by which MetS is recognized.
- A diagnosis of cancer is positively associated with the number of MetS components identified in each patient with MRD.
- MetS provides additional information to the already documented effect of increased inflammatory disease activity on carcinogenesis in MRD patients.

## Introduction

Metabolic syndrome (MetS) represents a cluster of cardio-metabolic disorders including obesity and visceral adiposity, insulin resistance, dyslipidemia, hyperglycemia, and hypertension. Several studies demonstrated the association between MetS and adverse prognosis in a lot of clinical settings, with special emphasis on those with high prevalence and public health impact such as cardiovascular diseases [1–3] and cancer [4–10]. Systemic chronic inflammation and the increased production of pro-inflammatory cytokines may favor the onset of MetS [11, 12]. This is the reason why patients with chronic inflammatory rheumatic and musculoskeletal diseases (RMD) such as rheumatoid arthritis (RA), psoriatic arthritis (PsA), and ankylosing spondylitis (AS) have increased prevalence of MetS [11, 13–15].

Besides MetS, RA/PsA/AS patients have a higher risk of developing cancer [3–7]. Some aspects of the relationship between chronic inflammatory RMD and malignancy have been recognized in the last years. Stimulation of B and T white cells by various antigens, overproduction of pro-inflammatory cytokines such as tumor necrosis factor (TNF-α) and/or interleukin (IL)1-IL6, exposure to environmental factors, and use of immunosuppressive drugs could all stimulate carcinogenesis in these subjects [3–7, 16, 17].

It is well known that MetS is closely related to the increased overall risks of cancer incidence and mortality in men and in women [8–10]. Furthermore, several studies on community populations found that patients with MetS had gender-related risk factors for total incident cancer and a gender-related predisposition for specific types of cancer [8–10]. However, although intuitive and rational, the association between MetS and cancer has never been analyzed in depth in patients with RMD, being still unclear whether, in this setting of patients, MetS represents an epiphenomenon of chronic inflammation leading to cancer or an independent risk factor for carcinogenesis.

Accordingly, we designed an ambispective (combination of retrospective and prospective) study aimed to assess prevalence/incidence and factors related to the development of cancer in a large cohort of patients suffering from RA/AS/PsA and to evaluate whether MetS and its components analyzed individually are associated with cancer (and which typologies of cancer) independently of traditional markers of inflammation and disease activity.

## Methods

### Study population

The study population comprised non-institutionalized subjects > 18 years of age in stable sinus rhythm with RA diagnosed according to the 2010 ACR/EULAR classification criteria [18], PsA and AS diagnosed by the CASPAR and the ASAS criteria as recently summarized by Rudwaleit and Taylor [19]. The design of the study was observational and ambispective (combination of retrospective and prospective) whereas malignancies were diagnosed before the recruitment and during the follow-up and considered all together into the final analysis. Patients were all referred to the Division of Rheumatology, Department of Medicine, University and Azienda Ospedaliera Universitaria Integrata of Verona (Italy). All patients visited between March 2014 and April 2016 who had neither history nor signs/symptoms and cardiovascular (CV) disease were selected for being involved in a CV primary prevention program and were recruited into this study. They underwent clinical, laboratory, and echocardiographic evaluations. All patients were closely followed up after recruitment. The period of observation ended on November 30, 2019. All patients gave written informed consent signing a specific institutional consent form; the study was approved by Ethical Committees of the Verona University and conforms to the ethical guidelines of the Declaration of Helsinki as revised in Brazil 2013.

### Definitions

MetS was diagnosed at recruitment (baseline evaluation) according to the ATPIII criteria (the National Cholesterol Education Program Adult Treatment Panel III) when 3 or more of the 5 conditions listed below were present [20]:

- Abdominal obesity defined as waist circumference > 102 cm in men and > 88 cm in women;
- Triglycerides ≥ 150 mg/dl;
- HDL cholesterol < 40 mg/dl for men and < 50 mg/dl for women;
- Blood pressure ≥ 130/≥ 85 mmHg;
- Fasting glucose ≥ 110 mg/dl (this condition was satisfied in patients with diabetes mellitus by definition).

Obesity was recognized when body mass index (BMI) ≥ 30 kg/m^2^. Dyslipidemia was defined as levels of total serum cholesterol > 190 mg/dl and or triglycerides > 150 mg/dl or pharmacologically treated high lipid serum levels. Renal function was assessed calculating the glomerular filtration rate (GFR) estimated by the CKD-EPI equation. We defined patients as biologic disease-modifying anti-rheumatic drugs (DMARDs) refractory on the date they had started their third class of biologic DMARDs before the enrolment into the study [21]. The degree of disease activity was evaluated by the clinical disease activity index (CDAI) in patients with RA and PsA [22] and with the Bath Ankylosing Spondylitis Disease Activity Index (BASDAI) in patients with AS [23]. RA and PsA patients with a CDAI > 10 [22] and AS patients with BASDAI > 7.3 [24] were defined as subjects with activated pattern of the disease having moderate-high disease activity. Left ventricular (LV) mass was calculated by means of transthoracic standard echocardiography using the Devereux’s formula and normalized for height to the 2.7 power. LV end-diastolic and end-systolic volumes were measured by the biplane method of disks from 2D apical 4 chamber + 2 chamber views and used to calculate ejection fraction, taken as index of LV systolic function.

### Statistical analysis

Data are reported as mean values ±1 standard deviation (medians and interquartile ranges for variables deviating from normality) or percentages. Unpaired Student’s test and *χ*^2^ statistics were used for descriptive statistics. Between-group comparisons of categorical and continuous variables were performed by *χ*^2^ test and analysis of variance with comparison between each group by Scheffè test for unequal sample, as appropriate or the Mann-Whitney non-parametrical test. Multivariate logistic regression analysis was performed to assess the factors independently associated with cancer. Thus, the factors associated with the event “cancer” were combined to build a clinical performance indicator which would better predict cancer than the factors considered separately. The ROC curve resulting from the performance indicator was compared using the z statistics with the curves resulting by each single factor of the performance indicator [25]. All analyses were performed using statistical package SPSS 19.0 (SPSS Inc. Chicago, Illinois), and statistical significance was identified by two-tailed *p* < 0.05.

## Results

### Study population

The study population included 474 subjects with RMD (244 RA, 96 AS, 134 PsA) predominantly middle-aged females with a long duration of chronic inflammatory rheumatic disease whose baseline characteristics are reported in Table 1. Hypertension and dyslipidemia were present in about half of patients; disease activity was moderate-high in one third of them. At enrollment, MetS was recognized in 76 patients (16%).

**Table 1:** Baseline characteristics of the study population divided in two subgroups according to the presence/absence of metabolic syndrome

Variables	Cancer No 428 patients	Cancer Yes 46 patients	***p***	Total study population 474 patients
Age (years)	57 ± 13	63 ± 11	0.001	58 ± 12
Female gender (%)	63	72	0.25	64
Body mass index (kg/height^2^)	25.9 ± 4.5	26.6 ± 4.8	0.31	26.0 ± 4.5
Waist circumference (cm)	92.4 ± 13.0	96.4 ± 12.4	0.04	93.1 ± 12.6
Obese (%)	15	24	0.14	16
Systolic blood pressure (mmHg)	130 ± 17	135 ± 18	0.04	131 ± 18
Diastolic blood pressure (mmHg)	82 ± 8	83 ± 9	0.23	82 ± 8
Hypertension (%)	44	65	0.006	47
Smoke (%)	33	29	0.99	32
Dyslipidemia (%)	55	67	0.10	57
Diabetes mellitus (%)	8	15	< 0.001	9
Metabolic syndrome (%)	6	29	< 0.001	16
eGFR (ml/min/m^2^*1.73)	94 ± 22	88 ± 24	0.13	93 ± 23
Hemoglobin (g/dl)	13.9 ± 1.4	13.6 ± 1.4	0.80	13.9 ± 1.4
Glycemia (mg/dl)	94 ± 22	106 ± 36	0.02	95 ± 25
Cholesterol HDL (mg/dl)	62 [45–80]	69 [47–91]	0.07	63 [46–83]
Cholesterol LDL (mg/dl)	123 [93–151]	120 [85–138]	0.58	121 [99–140]
Triglycerides (mg/dl)	119 [80–155]	125 [85–162]	0.66	120 [78–152]
C-reactive protein (mg/dl)	4.3 [2.8–7.5]	4.9 [3.5–8.9]	0.64	4.4 [2.9–7.8]
Rheumatoid factor positive (%) **	54	49	0.68	53
ACPA positive (%) **	52	47	0.80	51
Duration of disease (years)	12.2 ± 9.3	14.7 ± 9.8	0.67	12.6 ± 9.2
CDAI *	9.7 ± 8.9	13.7 ± 10.6	0.009	10.5 ± 8.4
DAS 28	2.4 ± 1.0	2.9 ± 1.0	0.08	2.5 ± 0.9
BASDAI	5.5 ± 3.3	6.0 ± 3.5	0.16	5.6 ± 3.5
Moderate/high disease activity (%)	32	55	0.005	33
LV mass (g/high ^2.7^)	43 ± 11	47 ± 10	0.04	44 ± 11
LVEF (%)	65 ± 6	67 ± 6	0.10	66 ± 6
**Medications**				
ACEi/ARBs (%)	27	44	0.02	29
Beta blockers (%)	15	28	0.04	16
Diuretics (%)	13	28	0.01	15
Calcium antagonists (%)	9	10	0.72	9
Statins (%)	18	42	< 0.001	20
Anti-platelets agents (*n*, %)	11	31	< 0.001	13
NSAIDs (%)	36	21	0.07	34
Methotrexate (%)	39	44	0.34	40
Hydroxychloroquine (%)	8	2	0.19	8
Corticosteroids (%)	36	31	0.25	35
Biologic DMARDs at enrolment (%)	66	63	0.66	66
Biologic DMARDs class			0.61	
Anti-TNFα (%) **	72	66		71
Anti-interleukin 6 (%)**	10	12		10
CTLA 4Ig (%)**	10	14		11
Anti-CD 20 (%)**	8	8		8
Biologic DMARDs refractory (%)	28	29	0.83	28

*ACEi* angiotensin-converting enzyme inhibitors, *ACPA* anti-cyclic citrullinated peptide antibodies, *ARB* angiotensin T1 receptor blockers, *CDAI* clinical disease activity index, *CD* cluster of differentiation, *CTLA* cytotoxic T lymphocyte antigen, *DMARDs* disease-modifying anti-rheumatic drugs, *ESR* erythrocyte sedimentation rate, *LVEF* left ventricular ejection fraction, *NSAIDs* non-steroidal anti-inflammatory drugs, *TNF* tissue necrosis factor

* % among patients with rheumatoid arthritis

** % among patients who were receiving biologic DMARDs

**Fig. 1:**
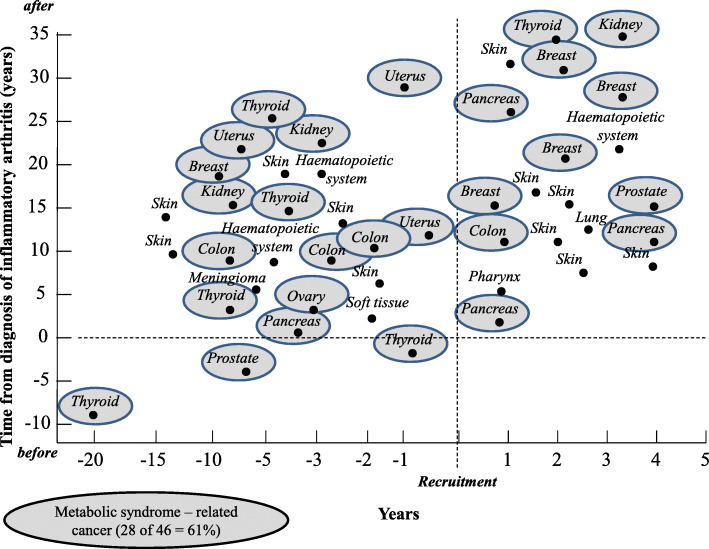
Typologies of cancer developed in the 474 study patients and the time of cancer diagnosis from the diagnosis of chronic inflammatory rheumatic disease and from enrollment. Malignancies traditionally associated with metabolic syndrome (MetS) are ringed by gray shadows

Duration of follow-up from recruitment was 42 [18–60] months. At the end of follow-up, patients with a diagnosis of cancer were 46 (9.7%). In 3 cases (7%), the diagnosis of cancer heralded the appearance of RA/AS/PsA disease; in 23 cases (50%), it was made from the diagnosis of RA/AS/PsA disease and our recruitment; and in 20 cases (43%), it was during the 42 months of follow-up. Figure 1 shows in detail the time from the diagnosis of cancer in relation to the time of the diagnosis of chronic inflammatory rheumatic arthritis and the enrollment and the typology of cancer.

Patients who had cancer were older with larger waist circumference, higher systolic blood pressure, prevalence of hypertension, diabetes, MetS, moderate/high disease activity, and LV mass than patients who had not. As expected, the former were receiving more frequently anti-hypertensive drugs, statins, and antiplatelet agents than the latter (Table 1). No pharmacological treatment for RA/AS/PsA disease was associated with the diagnosis of cancer. Also, the prevalence of biologic DMARDs refractory was similar between the two groups.

### Cancer and MetS

**Fig. 2:**
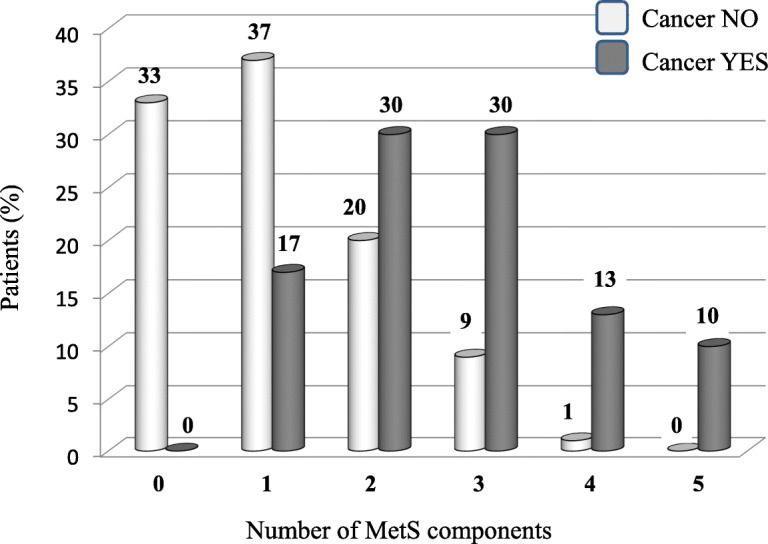
Distribution of patients with cancer according to the number of components of the metabolic syndrome (MetS) found in each patient

Considering the whole study population, cancer was diagnosed in 22 of 76 patients (29%) who had MetS (13 of 47 females = 28% and 9 of 29 males = 31%, *p* = 0.80) and in 24 of 398 patients (6%, *p* < 0.001) who had not. Among 46 patients who developed cancer, 28 (61%) had a malignancy traditionally associated with MetS (carcinoma of thyroid = 6, breast = 5, pancreas = 4, colon = 4, uterus = 3, kidney = 3, prostate = 2, ovary = 1). Considering the number of MetS components recognized in each study patient, cancer prevalently developed in subjects who had 2 to 5 MetS components (38 of 182 patients = 20.9%, corresponding to 83% of the total cases of cancer) while it was very rare among patients who had 0 to 1 MetS component (8 of 292 patients = 2.7%). Figure 2 shows in detail the distribution of patients with cancer according to the number of components of MetS found in each patient.

### Covariates of cancer

Variables significantly associated with cancer at univariate analysis are listed in the Table 2. Among these variables, age, MetS, moderate/high disease activity, and LV mass were included in the multivariate logistic regression model. This analysis revealed that the presence of MetS (OR 4.91 [CI 2.14–11.23], *p* < 0.001) together with older age and moderate-high disease activity were the states independently associated with cancer in our patients.

**Table 2:** Variables significantly associated with cancer: univariate and multivariate logistic regression analysis

Variables	Odds ratio	Confidence intervals	***p***	Odds ratio	Confidence intervals	***p***
Univariate analysis				Multivariate analysis		
Age (years)	1.05	1.02–1.08	< 0.001	1.04	1.01–1.08	0.03
Metabolic syndrome (%)	7.45	3.84–14.40	< 0.001	4.91	2.14–11.23	< 0.001
Moderate/high disease activity (%)	2.68	1.31–5.50	0.007	1.88	1.02–3.95	0.04
LV mass (g/high ^2.7^)	1.03	1.00–1.05	0.04	1.00	0.97–1.03	0.96
Hypertension (mmHg)	2.73	1.41–5.30	0.003	0.88	0.35–2.20	0.78
Cholesterol HDL (mg/dl)	1.01	0.99–1.03	0.16			
Triglycerides (mg/dl)	1.00	0.99–1.01	0.51			
Glycemia (mg/dl)	1.01	1.00–1.03	0.02			
Waist circumference (cm)	1.03	1.00–1.05	0.03			

### Associated factors of cancer

**Fig. 3:**
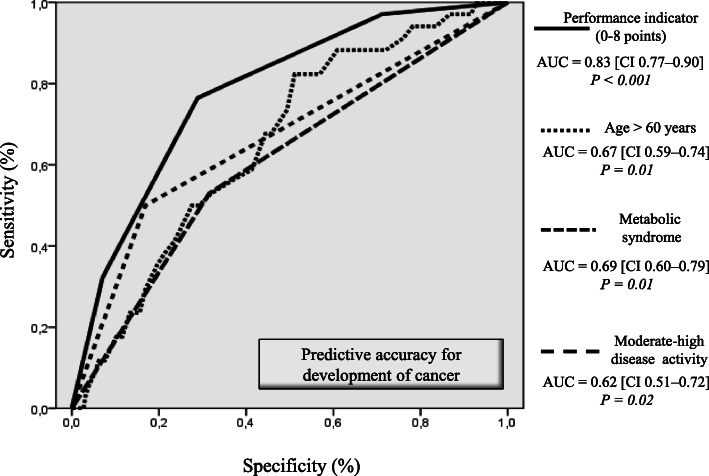
Performance indicator of the cancer prediction: ROC analyses of 3 variables associated with cancer (metabolic syndrome, age, moderate-high disease activity). Receiver operating characteristic (ROC) curve (derived by ROC analysis), area under the curve (AUC), and confidence intervals (CI) are shown. ROC curve built on the basis of the performance indicator for impaired sc-MFS at the end follow-up derived by the association of the 3 predictors is also shown

A performance indicator was built considering the 3 variables emerged by multiple logistic regression analysis as independent predictors of cancer. ROC curve analysis showed that the best cutoff values for age was 60 years. According to the Odds Ratios derived by multiple regression analysis, 5 points were assigned to patients with MetS, 2 points to those moderate-high disease activity, and 1 point to those with age > 60 years. Thus, the performance indicator ranged from 0 to 8 points, in case none or all 3 conditions were satisfied, respectively. Figure 3 shows that the performance indicator had an AUC clinically adequate (0.83 CI [0.77–0.90], specificity 89%, sensitivity 70% at the best cutoff = 5) which was compared by z statistics with the AUCs of the 3 prognosticators single-handed. The gap in accuracy for the prediction of cancer was statistically significant (*p* = 0.005 vs MetS, *p* = 0.001 vs age > 60 years, and *p* = 0.001 vs moderate-high disease activity). Finally, the observed event rate (development of cancer) was calculated according to the tertiles of the performance indicator. The patients belonging to the lowest tertile (including patients with points = 0–1) was 0.7% (1 of 146 patients), the median quartile (point 2–4) was 5.9% (10 of 170 patients), and the highest quartile of the performance indicator (points 5–8) was 22.2% (35 of 158 patients).

### Separate analysis of patients with RA, AS, and PsA

Since the extent of metabolic disease is very different in these diseases, despite the patient number is relatively small, we analyzed RA, AS, and PsA separately. Considering the whole study population (474 subjects), at the end of follow-up, patients with a diagnosis of cancer were 46 (10%): 27 (11%) belonged to RA sub-group, 16 (12%) to PsA, and 3 (3%) to AS (all *p* > 0.05). MetS was diagnosed in 76 of 474 patients (16%): 39 (16%) belonged to RA sub-group, 24 (18%) to PsA, and 13 (14%) to AS (all *p* > 0.05). Considering the whole study population, cancer was diagnosed in 22 of 76 patients (29%) who had MetS: 10 (26%) belonged to RA sub-group, 9 (37%) to PsA, and 3 (23%) to AS (all *p* > 0.05). Due to the very small sample size of each sub-group of patients, analyses of the factors associated with cancer in MetS provided unstable results and have not been showed.

## Discussion

Our study showed that (1) in subjects with RA/PsA/AS analyzed in a context of CV primary prevention program, the development of cancer is not uncommon and nearly two thirds of malignancies belong to those traditionally related to MetS; (2) more than a quarter of the patients in this study with MetS developed cancer overtime, so MetS represents the strongest risk factor independently of the various components by which MetS is recognized; (3) a diagnosis of cancer is positively associated with the number of MetS components identified in each patient; (4) MetS provides additional information to the already documented effect of increased inflammatory disease activity on carcinogenesis in these patients; and (5) information derived by logistic regression analysis allowed to build a simple performance indicator highly sensitive for cancer development.

The association between MetS and cancer was found in several clinical settings, with special emphasis on those with high prevalence and public health impact such as CV diseases [1–3, 8–10]. Stocks et al. analyzed seven European cohorts comprising 564.596 men and women with a mean age of 44 years followed-up for 12 years, showing increased risks for several cancers, in particular renal cell, liver, and colon cancer in men and endometrial and pancreatic cancer in women [8]. Nagel et al. reported that MetS score was related to 48% increased risk of Hodgkin’s lymphoma among women and BMI showed up as the most consistent risk factor, particularly in women [9]. Such associations were confirmed when a derived metabolic risk score of five metabolic factors was applied, in accordance with other investigations, mostly smaller studies, which have shown an increased risk for cancer (particularly of liver, colorectal and bladder cancer in men, and endometrial, pancreatic, breast postmenopausal, rectal and colorectal cancers in women) [10]. In our experience, these typologies of MetS-correlated cancer were evident in nearly two thirds of cases.

On the other side, chronic inflammatory arthritis is associated per se with an increased risk of cancer cause of chronic stimulation of B and T cells by various antigens, especially viruses, with consequent overproduction of pro-inflammatory/carcinogenic cytokines, environmental factors, and the use of immunosuppressive drugs [3–7].

We confirm this finding and add original information on the factors associated with MetS in patients with RA/PsA/AS. These subjects have higher magnitude of inflammation and a fivefold risk of developing cancer compared with counterparts without MetS. Furthermore, the risk of developing cancer overtime is tenfold higher in patients who have 2 or more MetS components than those who have less than 2 components. Collectively, our findings indicate that in patients with RA/PsA/AS, the relationship between higher magnitude of chronic inflammation and the risk of developing cancer is magnified by the presence of MetS and directly proportioned to the metabolic risk components.

Our data may lead to speculate that MetS would not merely be an epiphenomenon of the chronic inflammation leading to malignancy, but rather one of its contributory causes. The scientific community has clearly demonstrated that metabolic alterations induced by MetS strongly correlate with increased production of TNF-α, IL-6, and leptin, and with decreased production of adiponectin by the adipose tissue, where these molecules interfere with adipocyte metabolism [26–32]. Furthermore, it has been shown that MetS is associated with reduced rates of glucose oxidation and non-oxidative disposal, high rates of lipid oxidation, low energy expenditure, and impaired suppression of free-fatty acids [28]. Lower energy expenditure may indicate that subjects with this syndrome have central insulin resistance, a lower increase in meal-induced thermogenesis and thus a tendency to gain weight [31, 32]. Adipose tissue secretes a variety of molecules and adipocytokines, including TNF-α, IL-6, and adiponectin. However, MetS is associated with a high amount of intra-abdominal fat and, in this condition, the adiponectin levels are low. This is a worrisome condition, because adiponectin inhibits the expression of vascular cell adhesion molecules (i.e., VCAM-1), E-selectin, and has several anti-atherogenic and anti-inflammatory properties. Thus, hypo-adiponectinemia can be responsible for endothelial damage and sustains systemic chronic inflammatory state. All these conditions may represent reasons why patients with RA/AS/PsA and MetS are predisposed to cancer. These findings also suggest that MetS is both expression of the pathophysiologic mechanisms associated with RA/AS/PsA disease and/or a CV risk factor and a dominant indicator of frailty and of more advanced stage of inflammatory disease predisposing to adverse clinical events including cancer.

Our analysis showed that cancer was uncorrelated with any pharmacological treatment given to our patients overtime. Unfortunately, information on how traditional anti-inflammatory therapies for RA/AS/PsA including biologic DMARDs impact MetS and/or carcinogenesis are uncertain, as these drugs could both stimulate carcinogenesis through inflammation as well as favor tumor cell death [33]. Analyses from observational studies [34, 35] as well as from systematic reviews based upon trial data have widely found a neutral effect for most studies [36–40] but not all [41–43]. In a recent systematic review and meta-analysis of 74 randomized clinical trials comprising 29,214 patients, pooled results suggest that risk of cancer is increased in patients with rheumatologic diseases who are treated with interleukin inhibitors compared with placebo [40]. Even more recently, data from the U.S. Food and Drug Administration’s Adverse Event Reporting System gathered from the first quarter of 2004 to the end of 2015 were analyzed. The authors reported that methotrexate use was significantly associated with various malignancies in RA patients and that the concomitant use of biologic DMARDs further increased the risk of breast, ovarian, and lung cancers in methotrexate-treated patients [43].

Finally, analyzing the variables associated with cancer development overtime and putting together information on MetS, moderate-high disease activity, and age, we were able to arrange for a performance indicator which demonstrated a good accuracy and in particular a very high specificity, allowing to select the subgroup of RA/AS/PsA patients with a very low risk (< 1%) for cancer.

### Study limitations and strengths

Several limitations have to be underlined. Our data were collected by a single center which could lead to select RA/AS/PsA patients which might not represent the real world population of subjects with chronic inflammatory rheumatic diseases such as RA/AS/PsA. Secondly, we were not able to assess the degree of insulin resistance in our patients. Although reasonable and supported by indirect evidences which proved the close relationship between insulin resistance and MetS, information on patients taken individually is lacking. Thirdly, our findings do not allow definite judgments regarding the possible effects of some specific pharmacological treatment for RA/AS/PsA on the risk for cancer. Fourth, the presence of MetS was assessed only at baseline, so that changes overtime in body weight and lipid/glycemic profile could not be analyzed and considered in the analysis. Finally, as malignancies were diagnosed before the recruitment or during the follow-up, we cannot be sure that MetS steadily heralded or has some metabolic pathways common to cancer, like, as an example, both can be consequence of chronic inflammation. Otherwise, the study strengths consist of the complete nature of the data set, the prospective gathering of quite a lot of variables traditionally related to MetS and cancer, the quite long duration of follow-up, and the ability to have anamnestic and prospective information on all enrolled patients.

## Conclusions

In subjects with RA/PsA/AS analyzed in a CV primary prevention context, the development of cancer is not uncommon and nearly two thirds of malignancies belong to those traditionally related to MetS. In these patients, MetS seems to be closely associated with cancer independently of higher chronic inflammatory activity which remains an autonomous predisposing and/or influencing condition. A diagnosis of cancer overtime is positively associated with the number of MetS components identified in each patient. Our data allowed us to build a simple performance indicator highly sensitive for cancer development in RA/PsA/AS patients. In light of our results, an increasingly accurate assessment of MetS would be required in patients with RA/AS/PsA as potential measure of clinical outcomes (which could go beyond the role of simple CV risk factor) including the risk of cancer. Beside this, prospective investigations aimed to assess the potential favorable role of biologic DMARDs in the fight against cancer and/or the positive effects of pharmacological and non-pharmacological interventions to elude the development of MetS and its detrimental effects would be needed.

## Data Availability

Data sharing not applicable to this article; please contact the corresponding author for data requests.
